# Pain in adult and adolescent patients with 5q-associated Spinal Muscular Atrophy – an often underrated phenomenon

**DOI:** 10.1177/22143602251325773

**Published:** 2025-05-21

**Authors:** Lisa M Keipert, Claudia D Wurster, Zeljko Uzelac, Johannes Dorst, Joachim Schuster, Kurt Wollinsky, Albert Ludolph, Dorothée Lulé

**Affiliations:** 1 Department of Neurology, Ulm University, Ulm, Germany; 2 Institute of Human Genetics, Ulm University, Ulm, Germany; 3 German Center for Neurodegenerative Diseases (DZNE), Ulm, Germany; 4 Department of Anesthesiology, RKU, Ulm University, Ulm, Germany

**Keywords:** spinal muscular atrophy, SMA, pain, depression, depressiveness, quality of life

## Abstract

**Background:**

Spinal muscular atrophy (SMA) is a genetic disorder leading to progressive muscle weakness and atrophy. Pain in SMA may be the consequence of the underlying neuromuscular disease but has hardly been investigated so far.

**Objective:**

To assess pain in SMA and its interaction with patient's wellbeing.

**Methods:**

In a prospective, cross-sectional study design, 70 adult and adolescent SMA patients (median age 30 years, IQR 21–49 years, types I-IV) were assessed at the Department of Neurology, Ulm University hospital. Pain was evaluated with a self-adapted Pain Scale, depressiveness with the ALS-Depression-Inventory-12-Items (ADI-12) and global Quality of Life (gQoL) with the Anamnestic Comparative Self-Assessment (ACSA).

**Results:**

We found an intermittent frequency of pain in 80% in SMA patients with more than half of the patients experience pain at least once a week. The mean pain intensity score estimated by pain frequency and strength was 24 on a scale of 0 to 240, indicating a frequently appearing mild to moderate pain. Pain was mostly located in the lumbar spine, hip, and thoracic spine. The pain intensity score was independent from demographics (age, gender) or clinical parameters (SMA type, physical state), but, instead, it was associated to depressiveness. Depressiveness was more prevalent in older SMA patients. gQoL was rather independent from pain intensity or physical state.

**Conclusions:**

The study provides evidence for a prevalence of mild to moderate pain in 80% of adult and adolescent SMA patients. Pain was not simply caused by physical deficits and did not severely interfere with patients’ quality of life, but, instead, was closely interrelated with patients’ affective state.

## Introduction

Spinal muscular atrophy (SMA) is a rare neuromuscular disease with a frequency of 1:7.500 in newborns in Germany and 1:10.000 worldwide.^1,2^ It is caused by a homozygous deletion in the *SMN (survival motor neuron) 1* gene of exon 7 and/or 8 on chromosome 5q13.^3,4^ The deletion results in reduced production or loss of functional SMN protein, which causes a degeneration of the anterior horn cells in spinal cord and brainstem and, therefore, progressive, proximally accentuated and symmetric muscle weakness and atrophy. Secondary to muscular weakness pronounced changes in joints and spine such as contractures and scoliosis occur during the spontaneous course of disease. Patients are classified as type I to IV depending on age at disease onset, disease severity, and reaching certain motor milestones (sitting, standing, walking).^5,6^ Disease-modifying therapies have been available for this disease since 2017.^7–9^

Pain is a non-motor symptom experienced by patients with neuromuscular diseases. Studies indicate that the pain prevalence is approximately 70% in these patients.^10–13^ However, only few studies investigated pain in SMA patients only.^14–17^ Prevalences of pain in SMA ranging from 39% to 69% have been published.^12,14–17^ The lower extremities and the back were usually described as the main site of pain localization.^14,15^ However, the cause of the pain or the correlation of the pain with clinical characteristics or functional status has so far remained unclear.^16,17^

Depressiveness and global Quality of life (gQoL) as measures of patient's wellbeing have rarely been assessed in SMA patients, especially in the context of pain. Few studies show worse mental health in SMA patients with milder types and better physical function.^18,19^ Other studies show worse gQoL in SMA type I and II than in SMA type III patients.^20–22^ Pain is known to correlate with depressiveness, which again correlates with worse gQoL.^23–26^ Yet, there is limited understanding of pain's impact on mental health respectively the impact of the psychological state on pain.

Therefore, this study aims to evaluate pain intensity in adult and adolescent SMA patients the context of clinical phenotypes and its interaction with patient's depressiveness and gQoL.

## Materials and methods

### Design

This prospective cross-sectional study design was performed in adult and adolescent patients with SMA attending the SMA centre at the Department of Neurology at Ulm University hospital on a regular base, i.e., N = 56 for nusinersen treatment and N = 12 for risdiplam treatment every 4 to 6 months. These patients were on stable medication; N = 2 patients received no specific disease-modifying treatment. Data were assessed through a structured interview between November 2021 and July 2022, performed by a physician in training. The study was approved by the ethics committee of Ulm University (Approval Number 19/12, revised 23/03/2018). Written informed consent was obtained from all participants and/or their legal guardians.

### Pain

Pain was assessed using a self-adapted version of the German Pain Scale (GPS; following the Graded Chronic Pain Scale Items and Scoring (GCPS)^27^). The self-adapted Pain Scale recorded pain frequency (never = 0, intermittent = 1, once a month = 2, once a week = 3, several times a week = 4, daily = 5, all the time = 6), strength (on a scale from 1–10 in the morning, at noon, in the evening, at night), character (knocking, sharp, pulsating, pounding, ripping, piercing, cutting, dragging, burning, radiating), and localisation (head, cervical spine, thoracic spine, lumbar spine, shoulder, elbow, hand, upper extremity, hip, knee, foot, lower extremity). Also, currently and previously used pain medication, the use of other substances, and individual measures for pain relief as well as the use of alternative therapies were surveyed by an open question. Patients were allowed to choose more than one answer for pain character and localisation.

A pain intensity score was calculated from pain frequency and strength. All scores for pain strength were added up and multiplied by pain frequency. This created scores on a scale from 0 to 240 which represent the pain intensity score, with increasing scores indicating higher intensity or higher frequency, or both.

Furthermore, demographic and clinical factors as well as general condition (on a scale from 1 to 10) were recorded.

### Depressiveness and gQoL

Depressiveness was assessed using the ALS-Depression-Inventory-12-Items (ADI-12).^28^ This questionnaire, based on the Beck's depression inventory (BDI) and designed for use in patients with neurodegenerative diseases, consists of 12 questions with answers on a 4-point Likert scale that refer to the last two weeks and address mood, anhedonia, and energy. It does not evaluate any symptoms which may derive from the neurodegenerative disease itself.^28^ Scores range from 12 to 48 points, but as younger children were spared the question if “they often wished they were dead”, scores were transformed to percentages of the maximum score (ranging from 25 to 100%) to make them comparable. Scores between 23 and 28 points or between 47.9% and 58.3%, respectively, indicate symptoms of mild depression. For scores of 28 points (58.3%) or more a clinically relevant depression is suspected.^29^

GQoL was evaluated using Anamnestic Comparative Self-Assessment (ACSA), a self-anchored rating scale that is largely independent of age and other sociodemographic factors. ACSA is thus also applicable to young people.^30,31^ Patients were asked to name periods of their worst and best ever personally experienced gQoL to create a subjective scale. They were then asked to rate their overall QoL of the last two weeks on a scale ranging from −5 to +5 (−5 = worst ever personally experienced QoL; 0 = neutral state; + 5 = best ever personally experienced QoL).^32^

### Statistics

The statistical analysis was performed using SPSS 28.0.1.0. To test for normal distribution, the Kolmogorov-Smirnov-test was performed and, accordingly, median, IQR, minimum, and maximum are given for descriptive statistics. Spearman's rank correlation coefficient was used for correlations. Mann-Whitney-U-test was used for comparisons of two independent groups and ANOVA with Scheffé post-hoc test for multiple comparisons. To correct for covariance, we performed an ANCOVA. For statistical comparison of SMA types, type I and II were combined into group 1 (N = 35) whereas type III and IV were combined into group 2 (N = 35) due to account for the small numbers of patients in SMA I and IV. A threshold of *p* < 0.05 was adopted for statistical significance.

## Results

### Participants

A total of N = 70 SMA patients with a genetically confirmed deletion in exon 7 and/or 8 in the *SMN1* gene were included (type I: N = 6; type II: N = 29; type III: N = 33; type IV: N = 2). Of those, N = 59 were adult and N = 11 were adolescent with a median age of 30 years. 54.3% were male and 45.7% were female. N = 4 patients participated only in the pain, but not the mental health questionnaires (for further patient details see Tables 1 and 2).

**Table 1.** Demographic characteristics of SMA patients.

	Type I	Type II	Type III	Type IV	Total
N	6 (8.6%)	29 (41.4%)	33 (47.1%)	2 (2.9%)	70 (100.0%)
Age in years (Median, IQR)	20 (16.5, 24)	26 (17.0, 31)	40 (28.5, 51.5)	54.5 (53, -)	30 (21.0, 49)
Sex:					
Male	4 (66.7%)	15 (51.7%)	18 (54.5%)	1 (50.0%)	38 (54.3%)
Female	2 (33.3%)	14 (48.3)	15 (45.5%)	1 (50.0%)	32 (45.7%)

**Table 2.** Clinical characteristics indicating physical state of SMA patients.

	N	Percentage [%]
Scoliosis	53 / 70	75.7
Spondylodesis	24 / 70	34.4
Contractures	53 / 70	75.7
Fractures (ever in life)	36 / 70	51.4
Ventilation	30 / 70	41.4
Ventilation <8 h/day	13 / 30	43.3
Ventilation >8 h/day	17 / 30	56.7

### Pain

In total, 56 (80%) patients reported that they experienced at least intermittent pain in at least one body region. More than half of the patients (58.6%) reported pain at least once a week. 40 (57.1%) patients reported light pain, indicated by pain intensity between 1 and 4 on a scale from 0 to 10. Moderate pain (≥4 and < 7) was experienced by 12 patients (17.1%), while four patients (5.7%) reported severe pain, which corresponds to an average pain of ≥ 7. Only 14 (20%) patients never experienced pain. The pain intensity score showed a median intensity of 24 (scaled 0 to 240), which corresponds to a frequently appearing mild to moderate pain. Pain was mainly described as piercing (40%), dragging (30%) and radiating (28.6%) and was located mainly in the lumbar spine (47.1%), hip (28.6%), thoracic spine (24.3%), and lower extremities (22.9%). The current general condition on a scale from 0–10 showed a median of 8, which corresponds to an overall good condition.

Of the 80% of SMA patients who experienced pain, 42.9% regularly took pain medication, 7.1% used other substances such as tetrahydrocannabinol (THC) in form of medicinal cannabis for pain relief. 83.3% of those who reported the use of pain medication used non-opioid analgesics such as non-steroidal anti-inflammatory drugs (NSAID), 16.7% used opioids and co-analgesics. Seven patients (29.2%) took more than one medication. In addition to pain medication, SMA patients mostly applied changing position, movement, rest, heat, and massage for pain relief.

Pain intensity did not correlate with age (r = 0.092; *p* = 0.45) and gender (r = 0.016; *p* = 0.90). Furthermore, it did not differ significantly between groups 1 (SMA type I and II) and 2 (SMA type III and IV, *p* = 0.33). Pain frequency showed a significant correlation with the use of pain medication (r = 0.523, *p* < 0.001). Scoliosis (r = −0.094, *p* = 0.44), contractures (r = 0.014, *p* = 0.91) as well as history of fractures (r = 0.058, *p* = 0.64) and spondylodesis (r = −0.085, *p* = 0.48) showed no significant correlation with pain intensity. We also compared patients with pain to those without pain with regard to age (T = 0.76 *p* = 0.46) and gender (χ2 = 0.13 *p* = 0.77), but there was no significant difference between groups. In addition, we compared patients with and without pain with regard to scoliosis (χ2 = 0.18 *p* = 0.46), contractures (χ2 = 0.17 *p* = 0.46) as well as history of fractures (χ2 = 0.23 *p* = 0.43) and spondylodesis (χ2 = 0.57 *p* = 0.32), but again there was no significant difference between groups. When we compared patients with scoliosis and spondylodesis to those without spondylodesis and those without scoliosis, there was no difference for pain intensity (F = 0.51 *p* = 0.61), QoL (F = 0.52 *p* = 0.60) or pain severity (F = 0.21 *p* = 0.81).

### Depressiveness

**Figure 1.**
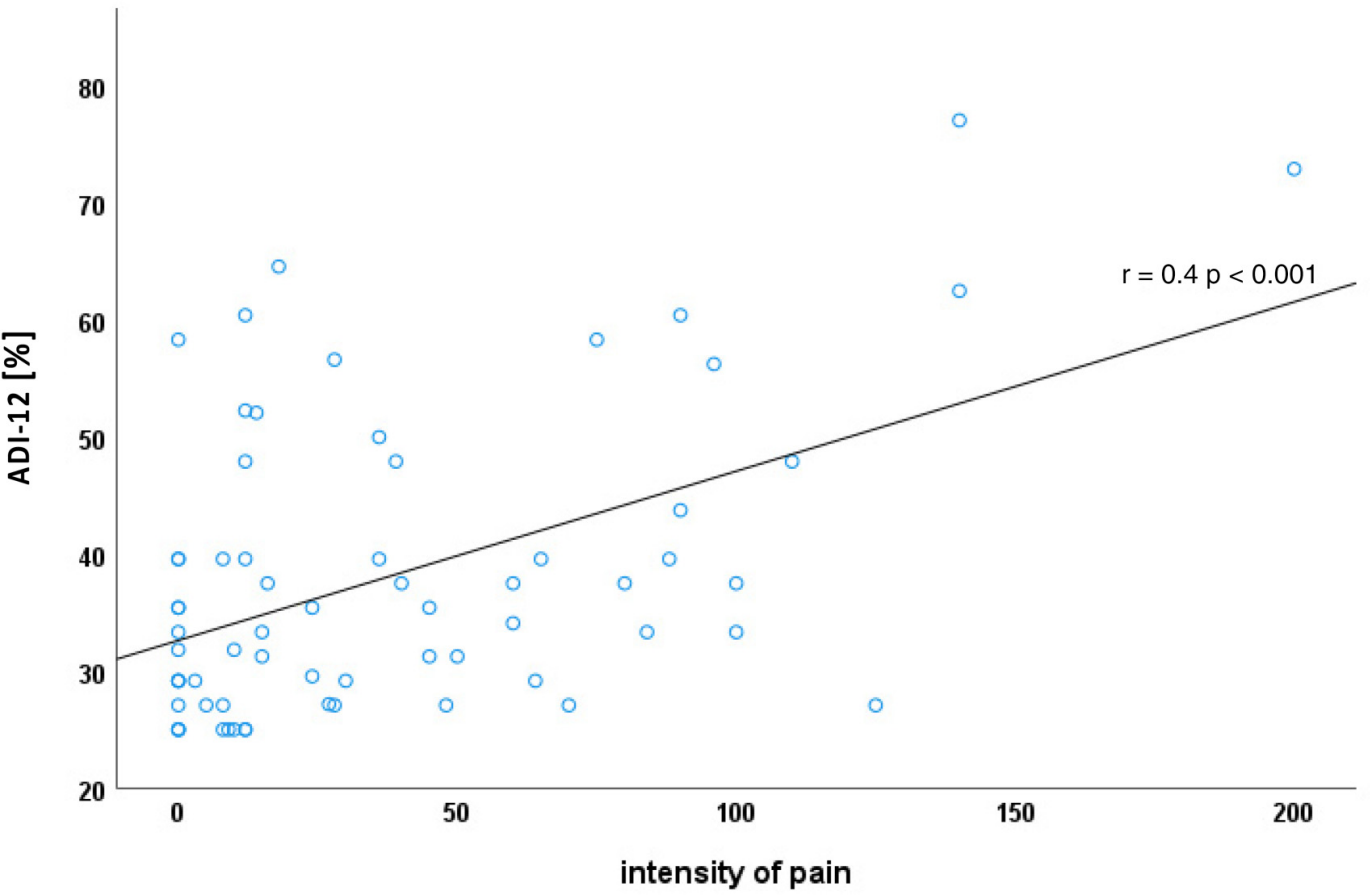
Association of pain intensity and level of depressiveness.

Legend: Pain intensity was assessed with the German Pain Scale (GPS) by adding up all pain strength scores for each point in time and multiplied by pain frequency, providing a score between 0 to 240. Depressiveness was assessed using the ALS-Depression-Inventory-12-Items (ADI-12), given as percentage of the maximum score (min 12 equals 25%, max 48 equals 100%). Spearman's rank correlation coefficient for correlations were used with a threshold of *p* < 0.05 for statistical significance.

In total, 50 (75.8%) SMA patients showed an ADI-12 score of < 47.9% and were thus considered not depressed in a clinically relevant way. Eight patients (12.1%) had a score between ≥ 47.9% and < 58.3% and thus showed mild depressive symptoms. Eight patients (12.1%) reached a score ≥ 58.3% and showed signs of clinically relevant depression. The ADI-12 score was positively correlated with the pain intensity score (the more pain, the higher the depressiveness score, r = 0.40, *p* < 0.001; Figure 1).

**Figure 2.**
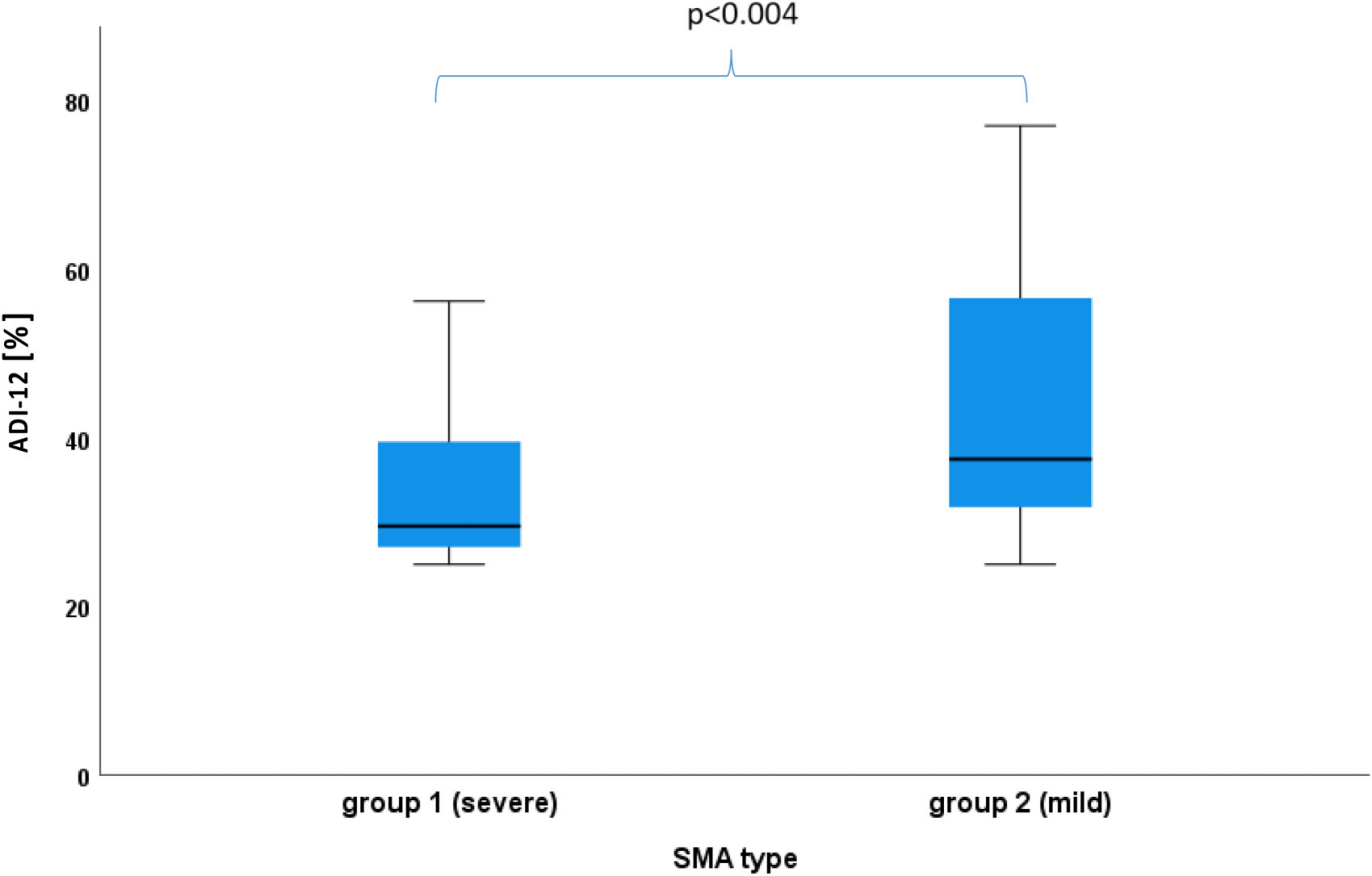
Difference in level of depressiveness between SMA subtype groups.

Legend: For comparison of SMA types, type I and II were combined into group 1 (N = 35) whereas type III and IV were combined into group 2 (N = 35). Depressiveness was assessed using the ALS-Depression-Inventory-12-Items (ADI-12), given as percentage of the maximum score (min 12 equals 25%, max 48 equals 100%). Mann-Whitney-U-test for two independent groups was used with a threshold of *p* < 0.05 for statistical significance.

The ADI-12 score was positively correlated with age (r = 0.382, *p* = 0.002), but not with sex (r = 0.70, *p* = 0.57). It differed significantly between group 1 and 2, whereby group 2 showed higher values (*p* = 0.004, Figure 2), but when the model was corrected for age, the difference between SMA groups no longer persisted (ANCOVA corrected for age with F = 2.80, *p* = 0.10).

Scoliosis (r = −0.188, *p* = 0.13), contractures (r = −0.227, *p* = 0.67) as well as history of fractures (r = 0.018, *p* = 0.89), and spondylodesis (r = −0.193, *p* = 0.12) showed no significant correlation with ADI-12.

### Gqol

The median ACSA score was 3, which corresponds to an overall good gQoL. There was no significant correlation between the ACSA score and pain intensity (r = −0.122, *p* = 0.33). The ACSA score showed a significant negative correlation with age (r = −0.416, *p* < 0.001), but not with sex (r = −0.049, *p* = 0.70). It did not differ between groups 1 and 2 (*p* = 0.16). No significant correlation was found between ACSA and SMA type (r = −0.199, *p* = 0.11). Scoliosis (r = 0.101, *p* = 0.42), contractures (r = 0.176, *p* = 0.16) as well as history of fractures (r = −0.142, *p* = 0.25) and spondylodesis (r = 0.016, *p* = 0.90) showed no significant correlation with ACSA. Additionally, a negative correlation was found between ADI-12 and ACSA (r = −0.614, *p* < 0.001). No significant difference was found between SMA patients with pain compared to those without pain regarding the ACSA score and thus gQoL (T = −0.38, *p* = 0.71).

## Discussion

### Pain

This study showed an at least intermittent pain prevalence of 80% in the assessed cohort of adult and adolescent SMA patients with more than half of the patients experiencing pain at least once a week. Although various SMA cohorts (regarding age or SMA type), different definitions (chronic, permanent, attacks) and measurement methods (e.g., self-designed patients’ questionnaire, visual analog scale (VAS), pressure algometer) of pain limit the comparison between studies, our data show an even more frequent occurrence of pain than previous studies which report prevalences of 39%,^15^ 40%,^14^ 43%,^16^ 55%^17^ and 69%,^12^ respectively.

In terms of pain intensity, more than half of the patients reported mild pain, while just over 20% of the patients in our study reported moderate to severe pain. Similar to our results, Uchio et al. reported chronic pain occurring in SMA also as usually mild.^14^

Pain in SMA patients has been reported to evolve secondary caused by scoliosis, contractures, fractures, and spondylodesis,^12,15,16,33^ but no association of these events and pain intensity was found in the current study. However, these comorbidities mainly affect the spine and large joints, e.g., the hip. Accordingly, patients often present with pain in these parts of the body. In the current study, the most frequently reported pain event was in the lumbar spine, hips, and thoracic spine, in line with existing data.^12,14,15^

There was no evidence for a difference in pain intensity between SMA I and II compared to SMA III and IV in our study, which was also demonstrated by other data,^14^ although different pathophysiological pain concepts, an “immobilization pain” in type II patients and an “exercised-induced pain” for SMA type III patients were discussed in this context.^14^ The influence of functional physical status on pain in SMA remains unclear, as it has been shown that patients with a better functional status are more likely to suffer from pain,^16^ but muscular weakness also contributes to the evolution of pain.^17^ Thus, this aspect needs further attention in future studies, but this may imply that other factors than SMA type or physical function may play a role in the subjective experience of pain intensity.

The use of pain medication (42.9%) in the present study was similar to other studies which reported frequencies of 51% and 35% respectively.^15,16^ These findings support the importance of assessing pain frequency and strength to initiate and adjust patient-centred pain therapy. In this context, it should also be mentioned that almost all patients in previous studies on pain in SMA (like our patients) received a disease-modifying therapy. However, the influence of these new therapies on pain cannot be adequately investigated with the cross-sectional design usually chosen and should therefore be the subject of further research.

### Depressiveness

Almost a quarter of the study population showed signs of at least mild depression. A study by Mix et al. showed similar findings, in which 19% of the patients showed signs of at least mild depression in ADI-12.^19^ In comparison, the lifetime prevalence of depression in Germany is approximately 10–12%.^34–36^ Therefore, SMA is associated with moderately increased depressiveness. Yet, it has to be considered that more than 75% of patients reported no signs of depressiveness, which highlights the lack of a fatalistic association of severe physical impairment and depressiveness.^37,38^ Previous studies have shown that pain and depression are interrelated: pain is often associated with a higher degree of depressiveness and depression is most common in patients with pain.^23,24^ Yet, this interrelation provides no proof of causality, and the question of cause and effect is unsolved . Individual studies have shown that SMA patients with severe pain suffer more from depression than patients with less severe pain. Also, affective state is most affected by pain.^12,13^ In line with this, there was a correlation between pain intensity and depressiveness in the current study.

Moreover, this study showed an association between a higher ADI-12 score and higher age of the SMA patients. It is striking that most of the older patients in this study were assigned to the SMA type III and IV group. However, after correction for age, we found no significant difference between the SMA groups, which may indicate that affective state is influenced less by functional status than by age. The prevalence of depression increases with age in Germany and is highest at about 45–64 years.^36,39^ Accordingly, current findings may also be seen in this context and may thus not exclusively be related to SMA.

### Global quality of life

The ACSA score indicated a generally good gQoL despite the physically severe disease. A study by Günther et al. showed a low prevalence of non-motor symptoms in SMA patients and a high degree of psychological resilience in most adult SMA patients.^40^ Similarly, a study by Fischer et al. found a level of psychological well-being comparable to healthy controls.^41^ Quality of life was not associated with the pain intensity score, moreover there was no difference between patients with pain to those without pain regarding gQoL. Instead, gQoL was negatively correlated with depressiveness and higher age.

### Limitations

This study is limited by the number of patients interviewed due to SMA being a rare disease and only limited numbers of SMA patients are treated in the Department of Neurology at the University Ulm. Thus, some results may have missed the threshold of statistical significance because the study population was too small. Yet, it is among the largest SMA cohorts, which has ever been investigated regarding mental well-being and pain. Furthermore, the majority of patients was diagnosed with type II or III, while patients with type I and IV were underrepresented, and also adult patients were overrepresented compared to paediatric patients. No healthy controls or patients with pain of any other origin were included. Therefore, future studies need to include these groups to further elucidate this aspect.

## Conclusion

This study found an intermittent frequency of pain in 80% of adult and adolescent SMA patients with more than half of the patients experiencing pain at least once a week. About 40% of patients suffering from pain took pain medication and additionally used other methods for pain relief such as changing position, movement, rest, heat, and massage. This illustrates that both pain assessment and adequate pain management are important for the comprehensive care of SMA patients, in addition to disease-specific therapy and treatment of respiratory, orthopaedic, and metabolic comorbidities. The correlation between pain intensity and depressiveness observed in this study highlights the increased presence of depression in patients suffering from pain. Yet, our data provide no proof of causality as depressed patients may as well be more likely to report pain. Depressiveness is most prevalent in older SMA patients and is linked to low gQoL. Summarizing, appropriate treatment of pain in addition to appropriate antidepressant medication in this subgroup, if necessary, should be an integral part of comprehensive treatment in SMA.
